# RJX Improves Wound Healing in Diabetic Rats

**DOI:** 10.3389/fendo.2022.874291

**Published:** 2022-06-02

**Authors:** Fatih M. Uckun, Cemal Orhan, Mehmet Tuzcu, Ali Said Durmus, Ibrahim H. Ozercan, Michael Volk, Kazim Sahin

**Affiliations:** ^1^ Drug Discovery Program, Reven Pharmaceuticals, Westminster, CO, United States; ^2^ Department of Developmental Therapeutics, Immunology, and Integrative Medicine, Ares Pharmaceuticals, St. Paul, MN, United States; ^3^ Department of Animal Nutrition, Faculty of Veterinary Medicine, Firat University, Elazig, Turkey; ^4^ Department of Biology, Faculty of Science, Firat University, Elazig, Turkey; ^5^ Department of Surgery Faculty of Veterinary Medicine, Firat University, Elazig, Turkey; ^6^ Department of Pathology Faculty of Medicine, Firat University, Elazig, Turkey

**Keywords:** COVID-19, RJX, pharmacology, pharmacodynamics, vitamins, nutrients, nutraceuticals, inflammation

## Abstract

**Background:**

We recently reported the clinical safety profile of RJX, a well-defined intravenous GMP-grade pharmaceutical formulation of anti-oxidant and anti-inflammatory vitamins as active ingredients, in a Phase 1 study in healthy volunteers (ClinicalTrials.gov Identifier: NCT03680105) (Uckun et al., Front. Pharmacol. 11, 594321. 10.3389/fphar.2020.594321). The primary objective of the present study was to examine the effects of GMP-grade RJX on wound and burn injury healing in diabetic rats.

**Methods:**

In the present study, a rat model of T2DM was used that employs HFD in combination with a single injection of STZ intraperitoneally (i.p) at a moderate dose level (45 mg/kg). Anesthetized diabetic rats underwent full-thickness skin excision on the back or were subjected to burn injury *via* a heated brass probe and then started on treatments with normal saline (NS = vehicle) or RJX administered *via* intraperitoneal injections for three weeks.

**Findings:**

Notably, diabetic rats treated with the 1.25 mL/kg or 2.5 mL/kg RJX (DM+RJX groups) rapidly healed their wounds as fast as non-diabetic control rats. Inflammatory cell infiltration in the dermis along with fibrin and cell debris on the epithelial layer persisted for up to 14 days in the DM+NS group but not in RJX-treated groups. The histopathological score of wound healing on days 7 and 14 was better in diabetic rats treated with RJX than diabetic rats treated with NS and comparable to the scores for non-diabetic healthy rats consistent with an accelerated healing process. The residual wound area of RJX-treated rats was significantly smaller than that of NS-treated diabetic rats at each evaluation time point (P<0.001). The accelerating effect of RJX on diabetic wound healing was dose-dependent. We obtained similar results in the burn injury model. Our results demonstrate that RJX – at a dose level >10-fold lower than its clinical maximum tolerated dose (MTD) – accelerates the healing of excision wounds as well burn injury in diabetic rats.

## Introduction

Skin integrity is critical for our defense against pathogens. Chronic skin ulcers, especially diabetic foot ulcers (DFU), affect millions of patients with diabetes mellitus (DM), especially those with sensory neuropathy, peripheral artery disease (PAD), and poorly controlled hyperglycemia, putting them at high risk for life-threatening infectious disease complications, including cellulitis, osteomyelitis, and sepsis (1–5). Such chronic non-healing wounds cause recurrent hospitalizations and ultimately require lower limb amputations with a very high risk of long-term morbidity and mortality (4, 6). While inflammation with an influx of neutrophils and macrophages into the affected skin segment is one of the pivotal early steps of wound healing and helps prevent superinfection of the wound, protracted and exacerbated inflammation in DM caused by inflammatory cytokines can delay the re-epithelization, reorganization of the extracellular matrix, as well as wound closure, and thereby undermine the timely tissue remodeling necessary to re-establish the integrity of the affected skin segment (7–9). An exaggerated neutrophil and macrophage influx during a prolonged inflammatory process with the release of reactive oxygen intermediates (ROI) can result in significant oxidative stress (OS) and cause inflammatory cell damage to keratinocytes and fibroblasts that are required for the formation of extracellular matrix and re-epithelization (10, 11). Further, increased production and release of MMPs and proteases also impede the assembly of extracellular matrix and wound closure (1, 5).

Developing effective strategies capable of reducing the prolonged inflammation and OS in diabetic wounds is an unmet medical need (12–16). Zhang et al. recently reported a global DFU prevalence of 6.3%, with the highest prevalence in North America at an estimated 13.0 % (USA: 13.0 %, Canada 14.8%) (17). Approximately one-third of the costs associated with DM is related to the management of DFUs and their complications (18, 19). The lifetime risk of a DFU for diabetic patients is approximately 15-20% which in part reflects the very high prevalence of peripheral arterial disease (PAD) in DM (19). The reported first-year probability of limb amputation in a newly diagnosed case of DFU is 34%, and the mortality rate is 5.5% with the severity of ulcer and presence of PAD as the key poor prognostic markers (20). In addition, patients with DM are at high risk for burn injuries due to their sensory neuropathy, leading to life-threatening infections and burn wound sepsis due to prolonged inflammation and delayed healing of the burned skin (21). Therefore, drugs capable of accelerating wound healing and facilitating rapid wound closure in DM patients are urgently needed (22–25).

RJX exhibited promising single-agent anti-inflammatory activity in a mouse model of fatal cytokine release syndrome (CRS) and acute respiratory distress syndrome (ARDS) (26). Preliminary evidence suggested that it may effectively reverse CRS and ARDS if administered after the onset of acute lung injury (ALI) in LPS-GalN challenged mice (26). The reported pharmacodynamic effects of RJX include (i) reduction of oxidative stress as measured by increased ascorbic acid concentration in serum and tissues, increased tissue activity levels of antioxidant defense enzymes superoxide dismutase (SOD), catalase (CAT), and glutathione peroxidase (GSH-Px), as well as reduction of tissue concentrations of malondialdehyde (MDA) (26, 27) and (ii) reduction of inflammatory biomarkers such as the serum and tissue levels of the proinflammatory cytokines interleukin 6 (IL-6) and tumor necrosis factor-alpha (TNF-α) (26). The proposed mode of action for RJX is a combined anti-oxidant and anti-inflammatory effect.

We recently reported the clinical safety profile of RJX in a Phase 1 study in healthy volunteers (ClinicalTrials.gov Identifier: NCT03680105) (26). The primary objective of the present study was to examine the effects of GMP-grade RJX on wound and burn injury healing in diabetic rats. Here, we demonstrate that RJX – at a dose level >10-fold lower than its clinical maximum tolerated dose (MTD) – accelerates the healing of excision wounds and burn injury in diabetic rats.

## Materials and Methods

### Rejuveinix

RJX is a GMP-grade pharmaceutical composition of anti-inflammatory and anti-oxidant vitamins (**Table S1** and **Supplemental Material**) (26, 27).

### Animals

For wound healing studies, 160 healthy female Wistar Albino rats (8 weeks old) were purchased from the Firat University Experimental Animal Center. The care of the animals was provided in accordance with the Guide for the Care and Use of Laboratory Animals. The study was approved by the Animal Care and Use Committee of Firat University (Skin Wound Project No. 01.06.2021-2254; Burn Injury Project No. 26.05.2021 - 2253). Eighty rats were used to evaluate the effects of RJX on diabetic wound healing, and 80 rats were used to assess the impact of RJX on the healing of diabetic burn injuries. Insulin sensitivity in rats is sex-dependent, with females exhibiting greater insulin sensitivity and susceptibility to diabetes (28). Therefore, female rats were used in our wound healing experiments. All rats had access to the assigned diet and water ad libitum during the study. Besides standard diet, high-fat diet (HFD) (Research Diets Inc., D12451) was used in specific experimental groups, as described herein below.

### Diabetic Wound Model

The combination of a high-fat diet (HFD) with streptozotocin (STZ) (i.p), a diabetogenic agent that causes mitochondrial dysfunction in the pancreatic β cells, causes hyperglycemia associated with hypertriglyceridemia and other features of human Type 2 Diabetes Mellitus (T2DM) (29).

The rats in the diabetic groups were fed an HFD for 4 weeks and then, on day 28, intraperitoneally injected with STZ at a moderate dose level (45 mg/kg, dissolved in 0.1 M sodium citrate buffer at pH 4.4) following 16 h of fasting, as previously reported (30, 31).

In experiments that employed the wound healing model, rats were randomly assigned to control (n=20) and diabetic wound groups (n=60). Rats in the healthy control group underwent full-thickness skin excision on the back (diameter length: 5 mm, with depth to the fascial layer; initial wound area = 19.625 mm^2^) and were treated with 2.5 ml/kg normal saline (NS) (i.e., an aqueous solution of 0.9% NaCl), i.p. x 21 days. Rats in the diabetic wound groups underwent full-thickness skin excision on the back (diameter length: 5 mm, with depth to the fascial layer) one week after STZ injection and were randomly assigned to 3 treatment groups (n=20/group): (1) DM+NS: 2.5 ml/kg/day i.p. NS as a vehicle; (2) DM + RJX 1.25 ml/kg/day i.p., 3) DM + RJX 2.5 ml/kg/day i.p. Skin excisions were performed under anesthesia [ketamine (85 mg/kg) + xylazine (6 mg/kg i.p.] on day 35 (7 days post STZ) and treatments with NS or RJX in the diabetic rats were initiated immediately after skin excision. All treatments were continued for 21 consecutive days (day 35-day 56).

### Diabetic Burn Model

In experiments that employed the burn injury model, rats were randomly assigned to control (n=20) and diabetic burn wound groups (n=60). Burn injury was induced in anesthetized rats [ketamine (85 mg/kg) + xylazine (6 mg/kg i.p.] on day 35 (7 days post-STZ) using a brass probe that was heated in boiling water and then placed on the shaved 1 cm diameter burn area on the back of the rats for 20 seconds. Rats in the healthy control group were treated with 2.5 ml/kg normal saline (NS), i.p. x 21 days. Rats in the diabetic burn injury groups underwent the skin burn procedure one week after STZ injection and randomly assigned to 3 treatment groups (n=20/group): (1) DM+NS: 2.5 ml/kg/day i.p. normal saline as vehicle; (2) DM + RJX 1.25 ml/kg/day i.p., 3) DM + RJX 2.5 ml/kg/day i.p. All treatments were continued for 21 consecutive days (day 35-day 56).

In both models, rats were monitored by taking photographs using a digital camera (Nikon D90, Tokyo, Japan) at multiple time points post-wound surgery/skin burn (days 3, 7, 14, and 21), and wound/burn injury healing was dynamically observed to calculate the healing rate. Wound/Burn healing rate = (original area-residual area)/original area x 100%. Wound closure = 100-[(original area-residual area)/original area x 100%]. Observation and evaluation of wound healing were carried out using Image J software (National Institutes of Health, Bethesda, MD, USA). Five random rats in each group were sacrificed on days 3, 7, 14, and 21 for histopathological evaluations and biomarker studies.

### Biomarker Studies

Tissue activity levels of superoxide dismutase (SOD), and tissue ascorbic acid levels were determined to examine the anti-oxidant effects of RJX, as previously reported (26, 27). We used commercial assay kits to determine tissue SOD levels as a measure of the oxidative stress status in the skin tissues of burn wounds, as previously reported (26). The activities of superoxide dismutase (SOD) were determined by quantitative enzyme-linked immunosorbent assays (ELISA, Bio-Tek Elx800 Universal Microplate Reader, Bio-Tek Instruments, Inc, Winooski, USA) using commercially available kits (Cayman Chemical, Ann Arbor, MI, USA) (26, 27). Liver tissue levels of ascorbic acid were measured by HPLC (26). Lipid peroxidation was determined as thiobarbituric acid reactive substances (TBARS), and malondialdehyde (MDA) levels in serum and tissue (MDA, nmol/ml serum, or nmol/g tissue) were measured by HPLC, as previously reported (26).

Levels of inflammatory cytokines interleukin-6 (IL-6) and tumor necrosis factor-alpha (TNF-α) in the skin wound and burn tissues were determined by Western blot analysis, as described (26). The insulin resistance index, was calculated by homeostasis model assessment of insulin resistance (HOMA-IR), as previously published (32). The HOMA-IR score was calculated as the product of the fasting insulin level (mU/L) and the fasting glucose level (mmol/L), divided by 76.4 for rats. We used a HOMA-IR cut-off value of 2.5 to define insulin resistance. Rats presenting with HOMA-IR ≥ 2.50 were considered insulin-resistant (32). Skin tissue hydroxyproline and total protein measurements were performed, as previously published (33).

### Histopathological Examination

After the elective sacrifice of rats by euthanasia after 3, 7, 14, and 21 days of treatment, full-thickness skin wound or burn area tissue samples were removed and subjected to histopathological examination. The tissue samples were fixed in 10 % neutral-buffered formalin solution, embedded in paraffin wax, cut into 5-μm-thick sections made from the center of each wound, and stained with hematoxylin-eosin and Masson’s trichrome stain, and examined by light microscopy. The histopathological scoring of skin excision wounds was performed in a blinded manner as described previously (34) (**Table S2**). Likewise, the histopathological scoring of skin burn areas was performed in a blinded manner as previously reported (35) (**Table S2**).

### Western Blotting

Skin tissue interleukin-6 (IL-6) and tumor necrosis factor-α (TNF-α) levels were evaluated by Western blotting in rat wound and burn samples as reported in detail earlier (26). The beta-actin antibody was used to control protein loading (Sigma). For each experimental condition, samples were examined in triplicate, and protein levels were assessed densitometrically using an image analysis system (ImageJ; National Institutes of Health, Bethesda, USA).

### Statistical Analysis

Standard statistical analyses included analysis of variance (ANOVA), nonparametric analysis of variance (Kruskal-Wallis), independent samples T-test, Shapiro-Wilk test, the Dunn’s multiple corporation test, and the Mann Whitney U test. We used the statistical programs IBM, SPPS Version 21 as well as GraphPad Prism version 8.0.

## Results

### Anti-oxidant, Anti-inflammatory, and Metabolic Effects of RJX in Rats on High-Fat Diet

RJX is well tolerated and exhibits anti-oxidant as well as anti-inflammatory activity in healthy rats on a standard diet (27). We first performed a preliminary study on 21 rats to determine if RJX has similar pharmacodynamic effects in rats on a high-fat diet (HFD). HFD diet and RJX were administered to groups of 7 rats for 12 consecutive weeks. HFD resulted in overweight of rats in the HFD+NS group, with the bodyweight differences between the control group on a standard diet and the HFD + NS group reaching statistical significance within 4 weeks (**Figure S1**). Control rats on a standard diet weighed 219.3±13.6 g at baseline and 358.9±15.5 g at 12 weeks. By comparison, NS-treated rats on HFD weighed 220.1±9.2 g at baseline and 443.7±25.3 g at 12 weeks. The difference at 12 weeks was highly significant (P<0.0001) (**Figure S1**). Administration of RJX throughout the 12-week study appeared to slow down the weight gain between 8 and 12 weeks (**Figure S1**). At 12 weeks, RJX-treated rats on HFD weighed 405.9±24.1 g, whereas NS-treated rats on HFD weighed 443.7±25.3 g (P=0.010) (**Figure S1**).

HFD-induced obesity of rats was associated with marked oxidative stress as documented by elevated levels of MDA as well as reduced levels of SOD in serum and liver specimens compared to control rats on a standard diet (P<0.0001 for all comparisons; **Figures S2A-E**). Treatment with RJX ameliorated the HFD-induced oxidative stress as evidenced by elevated tissue levels of Vitamin C, increased serum and tissue levels of SOD as well as reduced serum and tissue levels of MDA (Comparison of Groups HFD+RJX vs. HFD+NS: P<0.0001 for all biomarker comparisons) (**Figure S2**). HFD-induced obesity of rats also caused systemic inflammation with an elevation of serum CRP levels and a significant increase in the serum levels of the inflammatory cytokines IL-6 and TNF-α (**Figures S2F-H**). These adverse effects of HFD were mitigated by RJX (**Figure S2**). In the HFD+RJX group, CRP, TNF-α, and IL-6 levels decreased by 48.6%, 25.6%, and 35.3%, respectively, compared to the HFD+NS group (*p*<0.0001 for all).

HFD resulted in insulin resistance with significantly elevated fasting glucose levels and significantly elevated fasting insulin levels (**Figures S1C-E**; P<0.0001 for all comparisons between groups Control vs. HFD+NS). In view of its anti-oxidant and anti-inflammatory effects in rats on HFD, we next sought to determine if RJX could mitigate the HFD-associated insulin resistance. Administration of RJX throughout the 12-week study mitigated these metabolic consequences of HFD and prevented the development of insulin resistance (**Figure S1**). At 12 weeks, the serum levels of fasting glucose, fasting insulin (P<0.0001) as well as the HOMA-IR (P<0.001) values of RJX-treated rats in the HFD+RJX group were significantly lower than those of NS-treated rats in the HFD+NS group (P<0.0001, **Figure S1**).

### Obesity and Hyperglycemia in Wistar albino Rats Challenged With High-Fat Diet Plus Streptozotocin

Healthy control rats (N=20) used in the wound healing model weighed 196.1±2.0 g at baseline and 255.1 ± 2.0 g post regular diet on day 28. By comparison, the average body weights of the rats in the diabetic groups (N=60) were 192.1±3.6 at baseline and 291.7±5.8 post HFD on day 28. The difference in the day 28 body weights was statistically significant (**Figure S3**; P<0.0001).

Healthy control rats (N=20) used in the burn injury model weighed 204.9±1.7 g at baseline and 247.2±1.9 g post regular diet on day 28. By comparison, the average body weights of the rats in the diabetic groups (N=60) were 201.8 ± 1.0 g at baseline and 287.2 ± 1.6 g post HFD on day 28. The difference in the day 28 body weights was statistically significant (**Figure S4**; P<0.0001).

In the wound healing model, the fasting blood glucose levels in normal control rats remained within the normal range (5.71- 5.91 mmol/L) (**Figure S5A**). By contrast, all diabetic rats in the HFD/STZ-induced model of experimental T2DM, including those treated with RJX, had fasting blood glucose levels ≥13.88 mmol/L (i.e., 250 mg/dL) throughout the study. Similar results were observed in rats used in the burn healing model (**Figure S6A**). In both models, diabetic rats in the RJX treatment groups appeared to have lower blood glucose levels than NS-treated diabetic rats, especially on days 14 and 21 (P<0.001) (**Figures S5A**, **S6A**). Likewise, in both models, STZ-treated diabetic rats treated with normal saline (DM+NS group) had markedly reduced serum insulin levels compared to healthy non-diabetic control rats (**Figures S5B**, **S6B**). Furthermore, treatment with RJX 2.5 ml/kg/day i.p to diabetic rats resulted in a significant elevation in insulin levels compared to diabetic control animals (**Figures S5B**, **S6B**). These results demonstrate that all rats challenged with HFD plus STZ for the reported experiments in the diabetic wound healing and burn healing models had pronounced and sustained hyperglycemia throughout the study. They further suggest that targeting inflammation with RJX in this T2DM model may have some modest anti-diabetogenic effects.

### RJX Accelerates Diabetic Wound Healing

**Figure 1:**
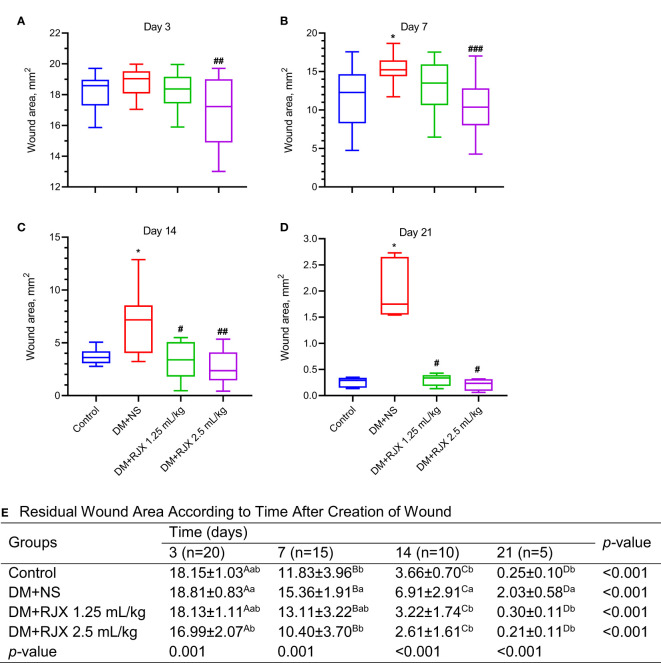
**(A–D)** RJX Accelerates Cutaneous Wound Healing in Diabetic, Obese Rats. **(A)** The Effects of Rejuveinix (RJX) on Wound Closure in Healing Diabetic Wounds. Wistar albino rats were treated with i.p injections of RJX (1.25 mL/kg and/or 2.5 mL/kg), or vehicle (NS). Except for untreated control rats (Control), each rat was fed a high-fat diet (HFD) for four weeks and injected a single dose of streptozotocin (STZ, 45 mg/kg i.p.) to induce diabetes (DM). At the end of 4 weeks, an experimental wound with a diameter of 5 mm (initial wound areas= 19.625 mm^2^) was created in all rats. The closure of the wound area was measured by digital camera until day 21. The depicted wound area data represent the median and min-max. The depicted wound area data represent the median and min-max. Statistical significance between groups is shown by *p<0.05 compared to the control group, and ^#^p<0.05; ^##^p<0.01 compared to the DM+NS group. ANOVA and Tukey’s *post-hoc* or Welch-ANOVA and Tamhane T2’s *post-hoc* test were used for comparing the results among different treatment groups. **(E)** Residual Wound Area in Diabetic Rats Treated with RJX vs NS. ^A,B,C,D^Means in a row with different superscripts are statistically different (p<0.05). ^a,b^Means in a column with different superscripts are statistically different (p<0.05). ANOVA and Tukey’s *post-hoc* or Welch-ANOVA and Tamhane T2’s *post-hoc* test were used for comparing the results among different treatment groups or different times. ^###^p<0.001 compared to the DM+NS group.

**Figure 2:**
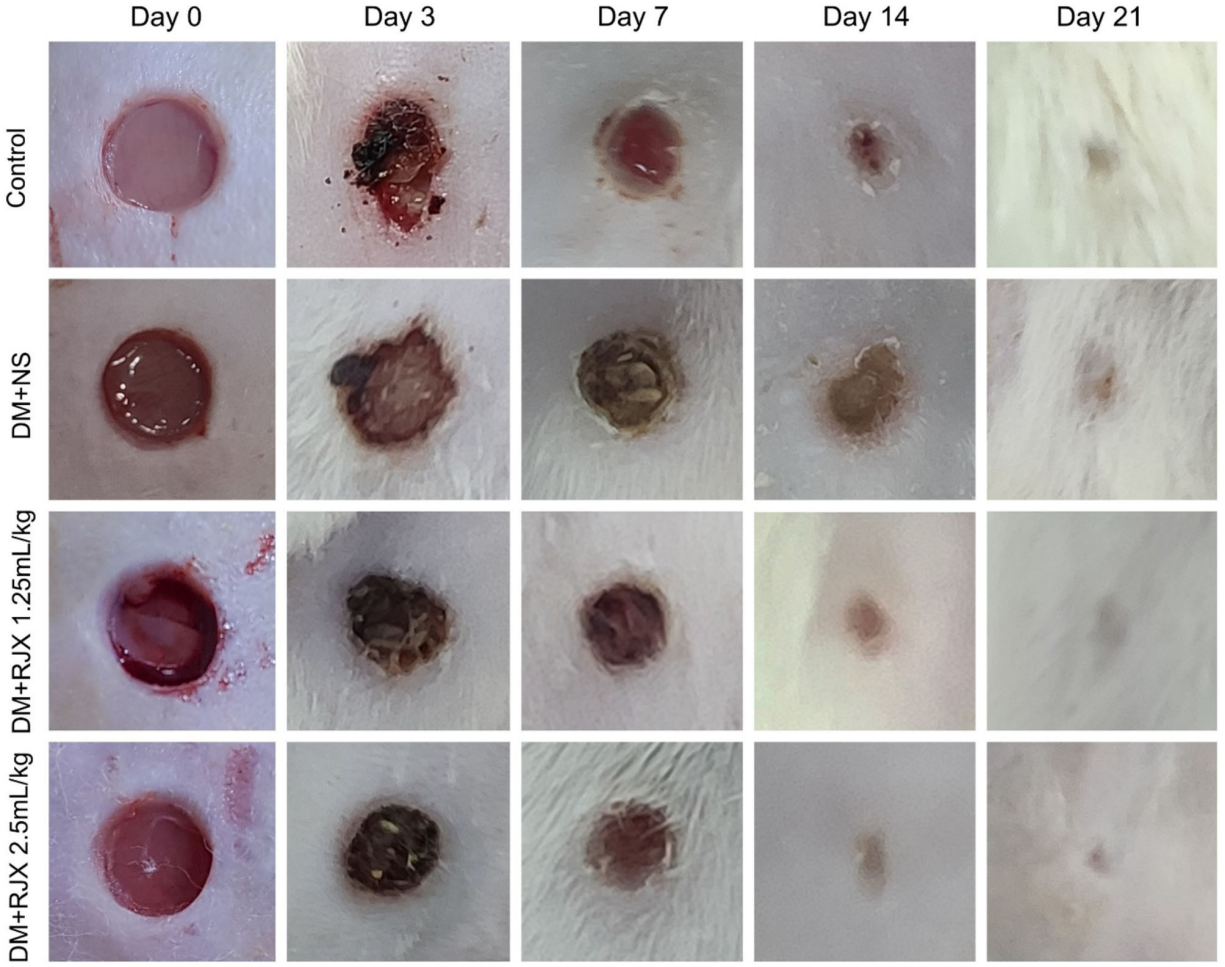
Effects of RJX on Cutaneous Wound Healing in Diabetic, Obese Rats. Representative wound bed images from rats in Figure 1. Healing of the wounds was significantly delayed in diabetic rats treated with NS compared to untreated non-diabetic control rats. Except for untreated control rats (Control), each rat was fed a high-fat diet (HFD) for 4 weeks and injected a single dose of streptozotocin (STZ, 45 mg/kg i.p.) to induce diabetes (DM). At the end of 4 weeks, an experimental wound with a diameter of 5 mm (initial wound areas=19.625 mm^2^) was formed in all rats. The macroscopic changes of the wound area were documented using a digital camera.

Macroscopically, non-diabetic control rats showed rapid healing of their cutaneous wounds within the 3-week observation period (**Figures 1**, **2**). The average wound area decreased from 18.15±1.03 mm^2^ at the beginning of the observation period to 3.66±0.70 mm^2^ (20.2% of initial wound area; 79.8% wound closure) on day 14 and 0.25±0.10 mm^2^ (1.4% of initial wound area; 98.6% wound closure) on day 21. By contrast, diabetic rats treated with NS had an average residual wound area of 6.91±2.91 mm2 (36.7% of initial wound area; 63.3% wound closure) on day 14, and 2.03±0.58 mm^2^ (10.8% of initial wound area; 89.2% wound closure) on day 21, which was approximately 8.1-fold larger than the residual wound area of non-diabetic control rats (**Figure 1**). By comparison, diabetic rats treated with the 1.25 mL/kg or 2.5 mL/kg RJX showed rapid healing of their wounds that was as fast as the wound healing in non-diabetic control rats (**Figure 1**). The residual wound area of RJX-treated rats was significantly smaller than that of NS-treated diabetic rats at each evaluation time point (P<0.001). The accelerating effect of RJX on diabetic wound healing was dose-dependent.

**Figure 3:**
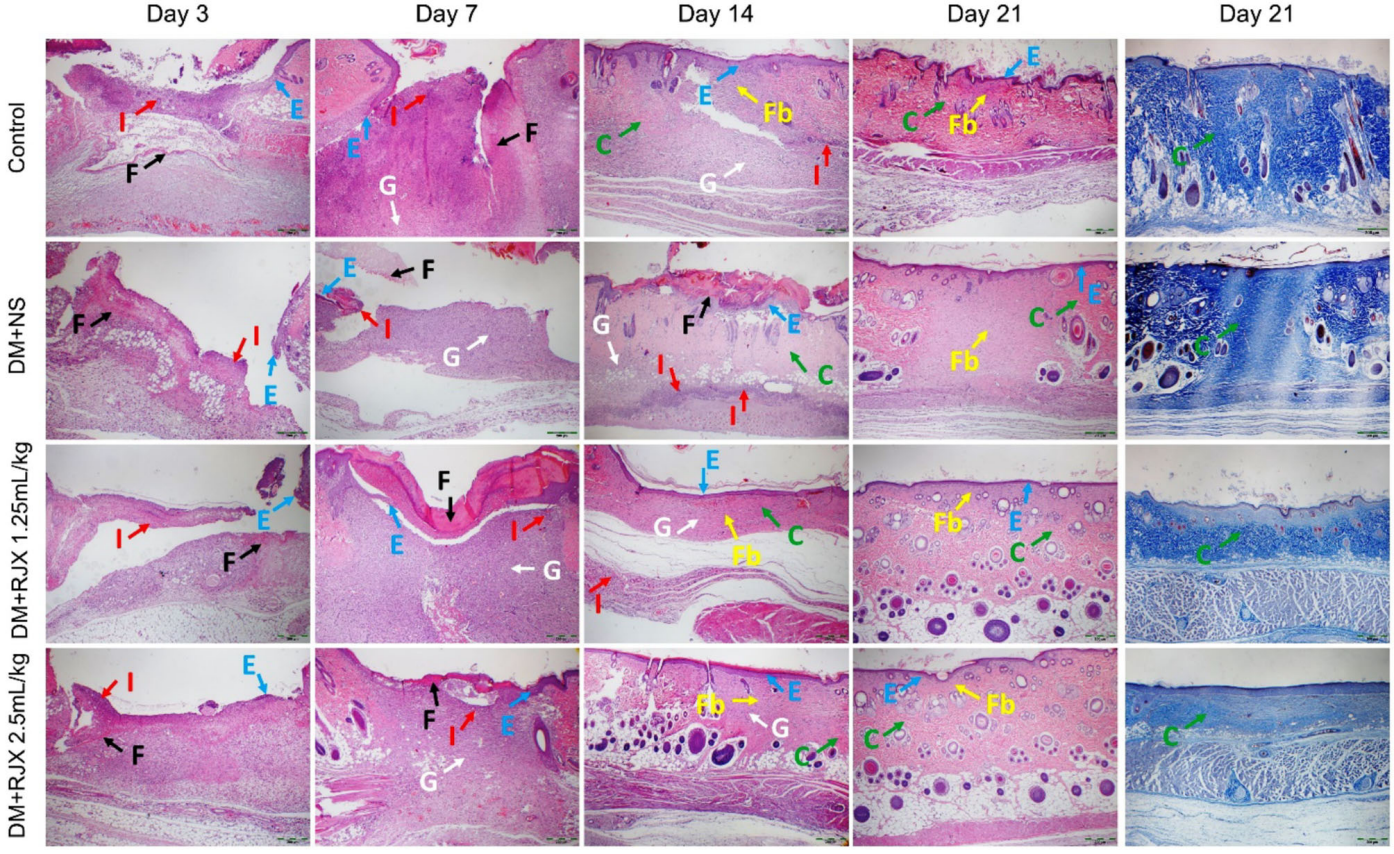
The Effects of Rejuveinix (RJX) on Histological Changes in Diabetic Wound Healing. Groups of 20 Wistar albino rats were treated with i.p injections of RJX (1.25 mL/kg and/or 2.5 mL/kg), or vehicle (NS). Except for untreated control rats (Control), each rat was fed a high-fat diet (HFD) for 4 weeks and injected a single dose of streptozotocin (STZ, 45 mg/kg i.p.) to induced diabetes (DM). At the end of 4 weeks, an experimental wound with a diameter of 5 mm was formed in all rats. On days 3, 7, 14, and 21, five rats in each group were randomly selected. Photomicrographs Demonstrating Rapid Re-epithelialization of Cutaneous Wounds In RJX-Treated Diabetic Rats. Columns 1-4: Blue arrow (E): Re-epithelialization; Red arrow (I): Inflammation; Black arrow (F): Fibrin, Fibrinogen; White arrow (G): Granulation; Green arrow (C): Collagen; Yellow arrow (Fb): Fibrosis. H&E X40. Column 5: Amplified Collagen Deposition in Skin Wounds of RJX-treated Diabetic Rats on Day 21. Masson’s trichrome staining. X40.

**Figure 4:**
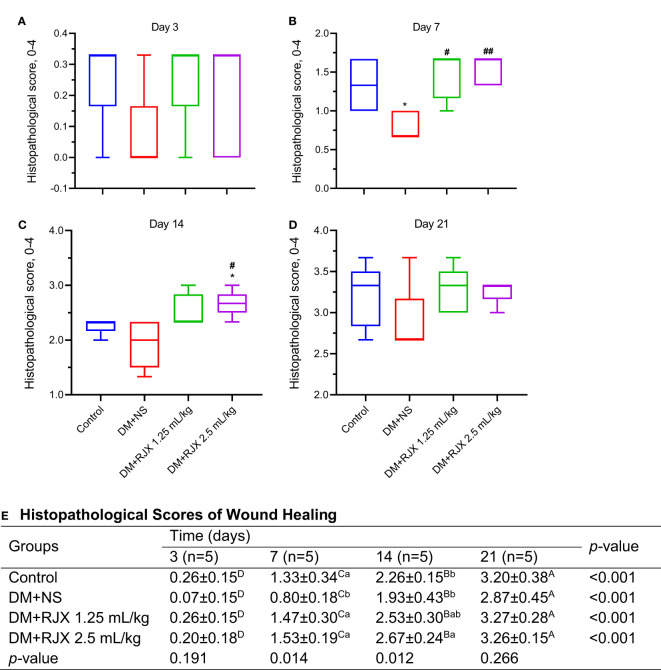
Histopathological Scores of Wound Healing by Presence of Diabetes and Treatment Group. **(A–D)** Histopathological scores for wound healing for the healthy control vs. diabetic rats. The depicted scores represent the median and min-max. Statistical significance between groups is shown by *p<0.05 as compared to the healthy control group, and ^#^p<0.05; ^##^p<0.01 as compared to the DM+NS group. Kruskal Wallis and Mann Whitney U tests were used for comparing the results among the different treatment groups. **(E)** Histopathological scoring system for wound healing. **(E)** Histopathological Scores of Wound Healing. On days 3, 7, 14, and 21, five rats in each group were randomly selected. The depicted histopathological score data represent the mean and standard deviation. ^A,B,C,D^Means in a row with different superscripts are statistically different (p<0.05). ^a,b^ Means in a column with different superscripts are statistically different (p<0.05). Kruskal Wallis and Mann Whitney U tests were used for comparing the results among different treatment groups or different times.

In the histopathological examination (**Figures 3**, **4**), early-onset epithelialization with fibrin, exudate, and inflammatory cell infiltration was detected in all groups on the 3rd day following skin excision. Rapid closure of the wound with a highly synchronized assembly of a fibrin clot, accumulation of granulation tissue, accelerated re-epithelization, and completion of the remodeling phase differentiated the microscopic changes of the healing wounds in diabetic rats treated with RJX from the late-onset re-epithelization of wounds in NS-treated diabetic rats (**Figure 3**). A significant increase was in re-epithelialization and fibroblast proliferation was evident as early as on the 7^th^ day in the control and RJX groups compared to the DM+NS group. On day 7, the histopathological score of wound healing was significantly lower for diabetic rats treated with NS than for healthy control rats, consistent with delayed wound healing (**Figure 4** **).** Inflammatory cell infiltration in the dermis along with fibrin and cell debris on the epithelial layer persisted for up to 14 days in the DM+NS group but not in the RJX-treated groups. The histopathological score of wound healing on days 7 and 14 was better in diabetic rats treated with 2.5 mL/kg RJX than for diabetic rats treated with NS and comparable to the scores for non-diabetic healthy rats consistent with an accelerated healing process (**Figure 4**).

On the 21st day, epithelialization was evident in the control and treatment groups, and mild fibroblast proliferation was observed under the epithelium. However, no inflammatory cells were found. Although epithelialization was complete in the DM+NS group, fibroblast proliferation under the epithelium covered a large area. Collagen regeneration was more pronounced in the DM + RJX groups on day 21 post-injury than in the DM + NS group (**Figure 3**). The organized collagen content of the granulation tissue in the skin wounds of diabetic animals is characteristically low, likely owing to persistent inflammation and elevated oxidative stress. Masson’s trichrome staining of the wound tissue specimens showed that the collagen deposition in the dermis and granulation tissue started earlier and was more pronounced in RJX-treated rats than in NS-treated rats, as reflected by more intense blue staining **(** **Figure 3** **).** By day 21, the collagen fibers in the healed skin covering the wounds of RJX-treated diabetic rats were arranged in bundles and positioned parallel to the new epidermis. These findings were consistent with the improved histopathological scores of the RJX treated groups (**Figure 4**).

**Figure 5:**
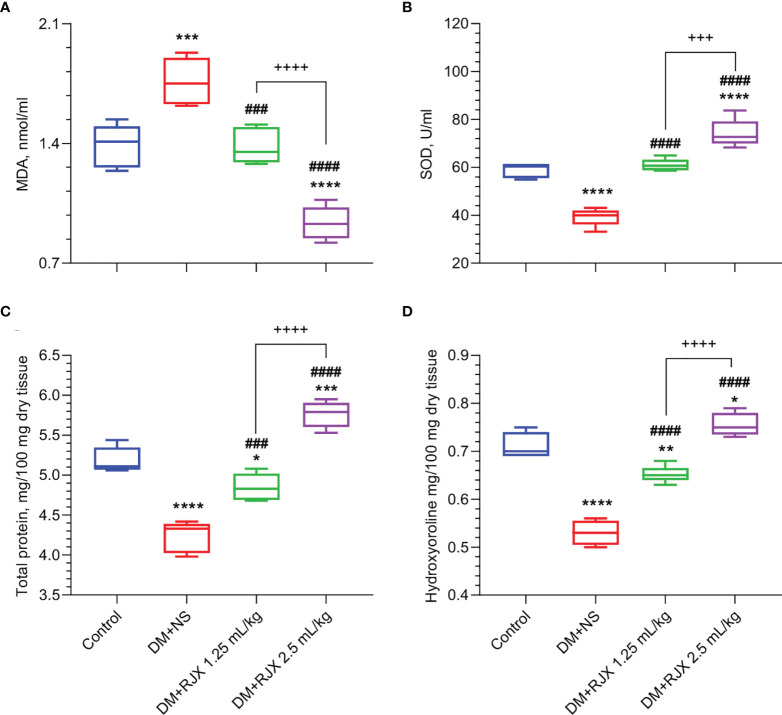
The Effects of Rejuveinix (RJX) on Malondialdehyde (MDA, **A**), Superoxide Dismutase (SOD, **B**), Skin Total Protein **(C)**, and Hydroxyproline **(D)** in Diabetic Wound Healing. Groups of 20 Wistar albino rats were treated with i.p injections of RJX (1.25 mL/kg and/or 2.5 mL/kg), or vehicle (NS). Except for untreated control rats (Control), each rat was fed a high-fat diet (HFD) for 4 weeks and injected a single dose of streptozotocin (STZ, 45 mg/kg i.p.) to induce diabetes (DM). At the end of 4 weeks, an experimental wound with a diameter of 5 mm was formed in all rats. On day 21, five rats (n=5) in each group were selected. The depicted Whisker plots represent the median and min-max values. ANOVA and Tukey’s *post-hoc* test were used for comparing the results among different treatment groups. Statistical significance between groups is shown by *p<0.05; **p<0.01; ***p<0.001; ****p<0.0001 as compared to control group, and ^###^p<0.001; ^####^p<0.0001 as compared to DM+NS group, and ^+++^ *p*<0.001; ^++++^ *p*<0.0001 pairwise comparisons between the groups.

**Figure 6:**
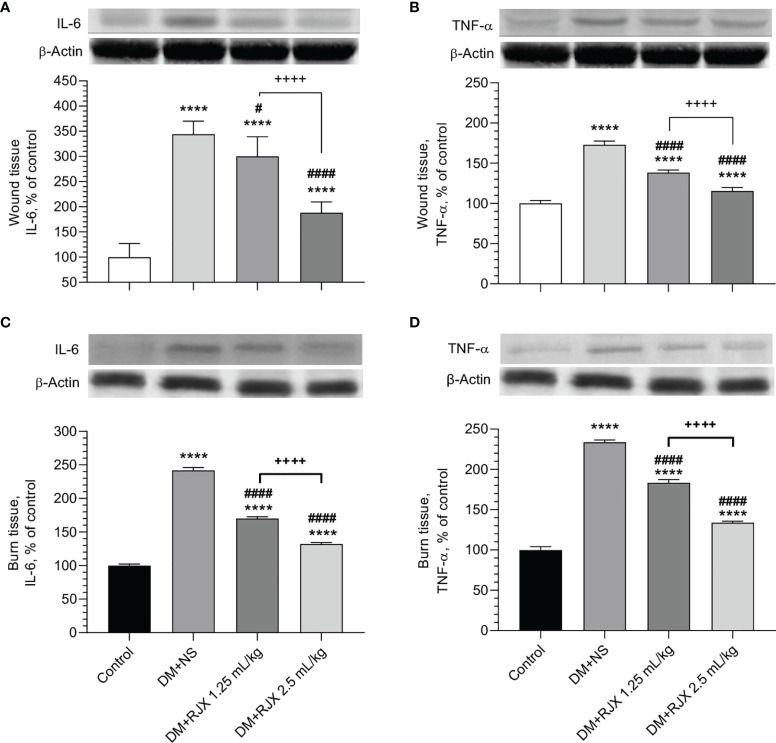
The Effects of Rejuveinix (RJX) on Interleukin-6 (IL-6, **A**) and Tumor Necrosis Factor-α (TNF-α, **B**) in Diabetic Wound Healing **(A, B)** or Burn Wounds **(C, D)** Induced Diabetic Rats. Groups of 20 Wistar albino rats were treated with i.p injections of RJX (1.25 mL/kg and/or 2.5 mL/kg), or vehicle (NS). Except for untreated control rats (Control), each rat was fed a high-fat diet (HFD) for 4 weeks and injected a single dose of streptozotocin (STZ, 45 mg/kg i.p.) to induce diabetes (DM). At the end of 4 weeks, an experimental wound with a diameter of 5 mm was formed in all rats. On day 21, five rats in each group were selected and skin tissues were removed and processed for Western blot analyses. The depicted bars represent the relative levels for IL-6 and TNF-a in skin wound or burn tissues of NS or RJX-treated diabetic rats in comparison to the levels for IL-6 and TNF-a in tissue specimens from healthy control rats (control). Results are expressed as percent of control with the expression level in the skin tissue samples from untreated healthy control rats taken as 100% for comparisons. Each bar represents the mean and standard deviation. Immunoblotting with an anti-actin antibody was used to ensure equal protein loading. ANOVA and Tukey’s *post-hoc* test were used for comparing the results among different treatment groups. Statistical significance between groups is shown by ****p<0.0001 as compared to control group, and ^#^p<0.05; ^####^p<0.0001 as compared to DM+NS group, and ^++++^ *p*<0.0001 pairwise comparisons between the groups.

Biomarker analyses performed on day 21 skin tissue specimens from NS-treated diabetic rats showed evidence of increased vulnerability due to oxidative stress, as measured by increased MDA and reduced SOD levels (**Figure 5**) and elevated levels of proinflammatory cytokines IL-6 and TNF-α (**Figure 6**) when compared to day 21 skin tissue specimens from healthy control rats.

As shown in **Figures 5A, B** the SOD levels in the healed day 21 wound tissues of diabetic rats were markedly elevated, and MDA levels measuring lipid peroxidation were significantly decreased in the RJX treatment groups. This anti-oxidant effect of RJX is consistent with the previously reported *in vivo* pharmacodynamic effects of RJX (26, 27). Likewise, day 21 skin tissue specimens from RJX-treated diabetic rats had significantly lower levels of IL-6 and TNF-a than the tissue specimens from NS-treated diabetic rats (**Figure 6A, B**). The observed effects of RJX on the tissue IL-6 and TNF-α levels are in accord with its published pharmacodynamic effects on these proinflammatory cytokines (26, 27). In agreement with the Masson’s trichrome staining data showing amplified collagen deposition, the levels of the major collagen component hydroxyproline, as well as the total protein levels, were significantly higher in the RJX treated diabetic rats than in NS-treated diabetic rats (**Figures 5C** **, D**).

### RJX Accelerates Diabetic Burn Healing

Macroscopically, non-diabetic control rats rapidly healed their burn wounds within the 3-week observation period (**Figure S7**). An accurate quantitative comparison of the wound area among different treatment groups was not feasible macroscopically due to necrotic tissue covering the burn surface of several rats at early time points and irregular scar formation at later time points. Histopathologically, irregular necrotic skin tissue remnants in the epidermis and dermis were observed on days 3 and 7 in all treatment groups. In non-diabetic control rats as well as RJX-treated diabetic rats, early re-epithelialization under the necrotic tissue was observed on days 3 and 7.

On day 14, marked infiltration with inflammatory cells, inflammatory neovascularization and fibroblast proliferation were evident in the epidermis and dermis of diabetic rats treated with NS, but not in diabetic rats treated with RJX (**Figure S8**). A significant increase in re-epithelialization was evident on the 14^th^ day in non-diabetic control and RJX-treated diabetic rats. While necrotic tissue disappeared on the surface in the treatment groups, epithelialization was complete, and significant fibroblast proliferation was observed under the epithelium in non-diabetic control rats as well as RJX-treated diabetic rats. Consequently, the day 14 histopathological burn healing scores of RJX-treated rats were significantly higher than those of NS treated diabetic rats (**Figure S9**). As in the wound healing model, the improved healing in RJX-treated rats was accompanied by higher SOD, lower MDA, higher protein, and higher hydroxyproline levels (**Figure S10**) as well as higher organized collagen content under the epithelium (**Figure S8**).

Biomarker analyses performed on day 21 burn tissue specimens from NS-treated diabetic rats showed evidence of increased vulnerability due to elevated levels of proinflammatory cytokines IL-6 and TNF-α (**Figure 6C, D**) when compared to day 21 skin tissue specimens from healthy control rats. Notably, the skin tissue specimens from RJX-treated diabetic rats had significantly lower levels of IL-6 and TNF-α than the tissue specimens from NS-treated diabetic rats (**Figure 6C, D**). The observed effects of RJX on the day 21 burn tissue IL-6 and TNF-α levels agree with its published *in vivo* pharmacodynamic effects on these proinflammatory cytokines (26).

## Discussion

The development of anti-inflammatory treatment platforms to improve wound healing in DM patients is an area of intense research focus (15, 35, 36). Strategies under evaluation include but are not limited to growth factors, oxygen therapy (16, 37), insulin (38, 39) and anti-DM medications with anti-inflammatory activities (25), adipose derived stem cells (ClinicalTrials.gov Identifier: NCT05095389), umbilical cord lining mesenchymal stem cells (ClinicalTrials.gov Identifier: NCT04104451), human amniotic membrane allograft (ClinicalTrials.gov Identifier: NCT02209051), Hydrogel sheet containing allogenic mesenchymal stem cells - ALLO-ASC-DFU Hydrogel sheet containing allogenic adipose-derived mesenchymal stem cells (ClinicalTrials.gov Identifier: NCT03754465), mesenchymal stem cell-derived exosomes (40), a synthetic hybrid-scale fiber matrix (ClinicalTrials.gov Identifier: NCT04918784), advanced topical wound dressings, natural extracts the Kalahari melon (Citrullus lanatus) seed oil (S26E) rich in unsaturated fatty acids (ClinicalTrials.gov Identifier: NCT04186377), topical nitric oxide releasing solution (NORS) for nitric oxide foot bath delivered as an adjunctive footbath treatment (ClinicalTrials.gov Identifier: NCT04755647), folinic acid (ClinicalTrials.gov Identifier: NCT04723134), topical application of genetically engineered probiotic bacteria expressing recombinant growth factors FGF2, IL4, bFGF, and CSF1(ClinicalTrials.gov Identifier: NCT04281992), synthetic hybrid-scale fiber matrix (Restrata^®^) with lyophilized acellular fish skin (Kerecis^®^ Omega3 Wound) (ClinicalTrials.gov Identifier: NCT04927702), portal, non-invasive, low level laser therapy device, that emits light in the near infrared (808nm) over an area of 1X4.5 cm^2^ with power output of 250mW, and energy dose of 5J/min. (ClinicalTrials.gov Identifier: NCT03687320), Altrazeal transforming powder dressing, a methacrylate-based powder dressing made from the same materials used in the production of contact lenses where the powder transforms into a moist, non-occlusive barrier that covers and protects the wound from exogenous bacteria while helping manage exudate through vapor transpiration) (ClinicalTrials.gov Identifier: NCT05046158), platelet-rich plasma-fibrin glue in combination with Vitamin E and C (ClinicalTrials.gov Identifier: NCT04315909), topical bacteriophage cocktail (ClinicalTrials.gov Identifier: NCT04803708), and macrophage-regulating drugs (41) (ClinicalTrials.gov Identifier: NCT01898923).

The primary objective of the present non-clinical proof-of-concept study was to evaluate the effects of RJX in two animal models of diabetic wound healing. In diabetic rats undergoing full thickness skin excision, RJX accelerated wound healing in a dose-dependent manner. Diabetic rats treated with the 1.25 mL/kg or 2.5 mL/kg RJX showed rapid healing of their wounds as fast as the wound healing in non-diabetic control rats. The macroscopic observation of the RJX-accelerated wound healing in diabetic rats was associated with a significant increase in re-epithelialization and markedly improved histopathological scores and fibroblast proliferation at early time points. RJX appeared to prevent the protracted inflammation phase that characterized the wounds of control diabetic rats treated with NS instead of RJX, augmented collagen synthesis and facilitated the tissue regeneration phase. Notably, the histopathological score of wound healing on days 7 and 14 was better in diabetic rats treated with 2.5 mL/kg RJX than diabetic rats treated with NS and comparable to the scores for non-diabetic healthy rats consistent with an accelerated healing process. Similarly, RJX-treated diabetic rats with a burn injury showed better histopathological burn healing scores than NS-treated diabetic rats. In both the wound healing model and burn injury healing model, wound specimens of RJX-treated diabetic rats showed higher SOD levels and lower MDA levels consistent with reduced oxidative stress as well as higher protein and higher hydroxyproline levels in accord with higher organized collagen content under the epithelium. These findings provide the preclinical proof of concept for the clinical development of RJX as an adjunct of the standard of care in the multi-modality management of DFU in patients with risk factors and its complications.

Hyperglycemia-triggered oxidative stress with amplified intracellular production of reactive oxygen species causing lipid peroxidation of cellular membranes and tissue level deficiency of anti-oxidant defense enzymes is one of the implicated contributors to nonhealing diabetic wounds (10, 11). Antioxidant-loaded hydrogels and SOD-loaded hydrogels have been proposed as platforms that could improve diabetic wound healing by decreasing ROS generation and oxidative stress in chronic wounds (14, 42). A recent study discovered significant micronutrient deficiencies in patients with DFUs (43). Sub-optimal systemic levels of Vitamin C characterize almost two-thirds of DM patients (43). Treatment with Vitamin C was reported to accelerate wound closure in diabetic mice (44). Another study established that Vitamin B12 deficiency in diabetic patients was associated with a 3-fold higher risk of DFU (45). Some of the glucose-lowering anti-diabetic medications (e.g., metformin, SGLT2 inhibitors, Dipeptidyl peptidase-4 (DPP-4) inhibitors) have been shown to exert anti-oxidant effects and have been explored as components of DFU management (25). Unfortunately, Metformin has been reported to cause vitamin B_12_ malabsorption leading to vitamin B_12_ deficiency as early as 3 months after initiation of therapy (46–48). In previous studies, we documented that RJX pharmacodynamically increases ascorbic acid as well as SOD levels in healthy rats. We also demonstrated in murine models of severe OS and systemic inflammation associated with reduced levels of the antioxidant enzymes SOD, CAT, and GSH-Px as well as ascorbic acid in lungs and liver that RJX decreased the tissue MDA levels, and normalized the levels of ascorbic acid and the antioxidant enzymes SOD, CAT, and GSH-Px (26). In this pre-clinical proof of concept study, we observed that the wound tissues (skin excision wounds as well as skin burn wounds) in RJX-treated diabetic rats had reduced OS as reflected by decreased MAD levels and increased levels of the anti-oxidant enzyme SOD. We, therefore, propose that RJX may help establish a balanced redox state in DFU and thereby improve their healing kinetics. As Vit-B12 is one of the active ingredients of RJX, its use may also mitigate the reported adverse consequences of inherent or Metformin-associated Vit-B12 deficiency in diabetic wounds (45–48).

Delayed wound healing contributes to an augmented risk of wound infection and severe sepsis with cardiovascular dysfunction, acute respiratory distress syndrome (ARDS), and fatal multi-organ failure in high-risk DM patients (21–24). RJX exhibits potent single-agent anti-inflammatory activity in the LPS-GalN model of fatal sepsis both in prophylactic settings for “prevention of sepsis” and in therapeutic settings for “treatment of sepsis” (26). RJX (i) profoundly decreased the inflammatory cytokine responses (IL-1β, IL-6, TNF-α, and TGF-β) to LPS-GalN (ii) mitigated the inflammatory tissue damage in the lungs and liver, and (iii) prevented a fatal outcome (26). Even when treatments were started after the onset of fulminant cytokine storm and systemic inflammation with severe oxidative stress, as well as very severe lung damage, a near-complete recovery of the inflammatory lung injury was achieved within 24 hours (28). In the open-label Phase 1 portion of an ongoing randomized, double-blind study in hospitalized COVID-19 patients with viral sepsis (ClinicalTrials.gov identifier: NCT04708340), none of the 13 patients treated with RJX plus standard of care developed a treatment-related DLT, SAE, or Grade 3-5 AEs. Nine (9) of the 12 evaluable patients, including 3 patients with hypoxemic respiratory failure, showed rapid clinical recovery (49). We hypothesize that the addition of RJX to the standard of care in high-risk DFU patients will not only shorten the time to clinical resolution of the limb-threatening complications but, because of its ability to reverse the cytokine-mediated multi-system inflammatory process in sepsis models, it may also reduce the risk of severe sepsis and sepsis-related mortality.

## Data Availability Statement

The original contributions presented in the study are included in the article/**Supplementary Material**. Further inquiries can be directed to the corresponding author.

## Ethics Statement

The animal study was reviewed and approved by The study was approved by the Animal Care and Use Committee of Firat University (Skin Wound Project No. 01.06.2021-2254; Burn Injury Project No. 26.05.2021 - 2253)

## Author Contributions

Each author has made significant and substantive contributions to the study, reviewed and revised the manuscript, provided final approval for submission of the final version. FMU and KS conceived the study, designed the evaluations reported in this paper, directed the data compilation and analysis, analyzed the data, and prepared the initial draft of the manuscript. KS, AD, MT and CO collected the non-clinical data and performed their analysis. IO performed the necropsies and histopathologic examinations on mice. All authors contributed to the article and approved the submitted version.

## Funding

This study received funding from Reven Pharmaceuticals, LLC, a wholly-owned subsidiary of Reven Holdings Inc. The funder was not involved in the study design, collection, analysis, interpretation of data, the writing of this article or the decision to submit it for publication. Among the authors, FMU, who participated in the analysis and decision to submit the manuscript for publication, was a consultant of Reven Pharmaceuticals. All authors declare no other competing interests.

## Conflict of Interest

Author FMU is employed by Ares Pharmaceuticals, and he served as a consultant for Reven Pharmaceuticals. FMU participated in the analysis of data as well as manuscript submission decisions. MV serves as a consultant for Reven Pharmaceuticals.

The remaining authors declare that the research was conducted in the absence of any commercial or financial relationships that could be construed as a potential conflict of interest.

## Publisher’s Note

All claims expressed in this article are solely those of the authors and do not necessarily represent those of their affiliated organizations, or those of the publisher, the editors and the reviewers. Any product that may be evaluated in this article, or claim that may be made by its manufacturer, is not guaranteed or endorsed by the publisher.
