# Supplementary material for: RJX Improves Wound Healing in Diabetic Rats
Source: Front Endocrinol (Lausanne). 2022 Jun 2;13:874291. doi: 10.3389/fendo.2022.874291 (PMC9201994; doi:10.3389/fendo.2022.874291)
Supplement: Supplementary file 1 [file DataSheet_1.docx]

Supplemental Tables and Figures

Table S1. Quantitative Composition of Investigational Product

1. RJX Vial A: Lot PPP.18.1051

| **Lot #PPP.18.1051** | | |  |  |
| --- | --- | --- | --- | --- |
| **Test** | **Specification** | | **Test Results** | |
| **Appearance** | Brownish-orange/reddish-orange | | conforms | |
| **pH** | 2.2 - 3.8 | | 3.0 | |
| **Sterility** | USP <71> | | Sterile | |
| **Endotoxin** | USP <85>, NMT 17.5 EU/mL – MVD = 1:3500 | | <2.5000 EU/mL | |
| **Particulate Matter** | USP <788>, The average number of particles present in the units tested does not exceed 6000 per container ≥ 10 μm and does not exceed 600 per container ≥ 25 μm | | ≥ 10 µm: 15 particles/container;  ≥ 25 µm: 0 particles/container | |
| **Assay** | Lower Limit (% content by weight) | Upper Limit (% content by weight) | Measured % content | % of Specified Target Content |
| **Ascorbic Acid** | 8.094% | 9.893% | 9.011 | 100.2 |
| **Niacinamide** | 1.069% | 1.307% | 1.186 | 99.8 |
| **Pyridoxine HCl** | 1.069% | 1.307% | 1.191 | 100.3 |
| **Riboflavin 5' Phosphate** | 0.023% | 0.028% | 0.025 | 98.8 |
| **Thiamine HCl** | 0.570% | 0.697% | 0.643 | 101.5 |
| **Calcium Pantothenate** | 0.026% | 0.032% | 0.028 | 95.6 |
| **Cyanocobalamin** | 0.017% | 0.021% | 0.019 | 98.4 |
| **Magnesium Sulfate** | 7.272% | 8.888% | 8.176 | 101.2 |

1. **RJX Vial B – Lot # PPP.18.998**

| **Lot #PPP.18.998** | | | |  | |
| --- | --- | --- | --- | --- | --- |
| **Test** | **Specification** | | | **Test Results** | |
| **Appearance** | Clear, colorless, free of particulate | | | conforms | |
| **pH** | 6.2 – 8.4 | | | 8.3 | |
| **Sterility** | USP <71> | | | Sterile | |
| **Endotoxin** | USP <85>, NMT 17.5 EU/mL – MVD = 1:3500 | | | <0.0500 EU/mL | |
| **Particulate Matter** | USP <788>, The average number of particles present in the units tested does not exceed 6000 per container ≥ 10 μm and does not exceed 600 per container ≥ 25 μm | | | ≥ 10 µm: 10 particles/container;  ≥ 25 µm: 3 particles/container | |
| **Assay** | Target (% content by weight) | Lower Limit (% content by weight) | Upper Limit (% content by weight) | Measured % content | % of Specified Target Content |
| **Sodium Bicarbonate** | 8.40 | 7.56 | 9.24 | 8.80 | 104.8 |

NT = not tested at this interval

**Table S2. Histopathological Scoring System for Evaluation of Wound Healing**

**A. Skin Excision Wounds**

| **Score** | **Re-epithelialization** | **Granulation tissue formation** | **Collagen organization** |
| --- | --- | --- | --- |
| 0 | None | None | None |
| 1 | Migrating | Hypo cellular with few vessels | Trace |
| 2 | Partial stratum corneum | Many vessels and some cells | Slight |
| 3 | Hypertrophic | Many fibroblasts, some fibers | Moderate |
| 4 | Complete and normal | More fibers few cells | Marked |

**B. Burn Injury Wounds**

| Scores | Parameters |
| --- | --- |
| 0-1 | There was loss of dermal component, lot of necrotic tissue, lymphocytic infiltration hyperemia in hypoderm |
| 1-2 | There was necrotic tissue, inflammatory cells and onset of new blood vessel formation and fibroblasts |
| 2-3 | There was fibroblast proliferation, accumulation of collagen, new blood vessel formation and inflammatory cells |
| 3-4 | There was proliferation of fibroblast, accumulation of collagen, new blood vessel appearance, few inflammatory cells and newly formed epithelial tissue |
| 4-5 | There was marked epithelialization and scar tissue formation |
| 5-6 | There was complete epithelialization, regeneration of hair follicles, sebaceous gland, and scar tissue of the burn |

**Figure S1. The effect of Rejuveinix (RJX) on Body Weight Change (A), Final Body Weight (B), Glucose (C), Insulin (D), and HOMA-IR (E) in rats fed high-fat diet (HFD). The rats (n=7) were fed either Control standard diet or an HFD (45% of calories as fat) or an HFD administered with RJX 4.2** **mL/kg via intraperitoneal injection (HFD+RJX) RJX were given for 12 weeks. [A]** The depicted Point-line plots represent the mean and standard error (SE). **[A]** ANOVA and Tukey’s post-hoc test were used for comparing the results among different treatment groups. Statistical significance between groups is shown by * *p<*0.05; ** *p<*0.01; **** *p<*0.0001 as compared to control group. **[B-E]** The depicted Whisker plots represent the median and values. ANOVA and Tukey’s post-hoc test were used for comparing the results among different treatment groups. Statistical significance between groups is shown by ** *p<*0.01; **** *p<*0.0001 as compared to control group, and # *p<*0.05; ### *p<*0.001 as compared to HFD group.

**Figure S2. Effects of RJX on HFD-associated Oxidative Stress and Systemic Inflammation.** Groups of 7 rats were fed either standard diet (Control) or HFD. Rats on HFD were treated with intraperitoneal injections of 4.2 mL/kg NS (vehicle control) or RJX for 12 weeks. The depicted Whisker plots represent the median and values for the serum MDA (Panel A), SOD (Panel B), liver MDA (Panel C), liver SOD (Panel D), liver ascorbic acid (Panel E), and CRP (Panel F), TNFα (Panel G), and IL-6 (Panel H). ANOVA and Tukey’s post-hoc test were used for comparing the results among different treatment groups. Statistical significance between groups is shown by **** *p<*0.0001 as compared to control group, and #### *p<*0.0001 as compared to HFD+NS group.

**Figure S3. Body Weight Change of Rats in Different Treatment Groups in the Diabetic Wound Healing Model.** Groups of 20 Wistar albino rats were treated with i.p injections of RJX (1.25 mL/kg and/or 2.5 mL/kg), or vehicle (NS). Except for untreated control rats (Control), each rat was fed a high-fat diet (HFD) for 4 weeks and injected a single dose of streptozotocin (STZ, 45 mg/kg i.p.) to induce diabetes (DM). At the end of 4 weeks, an experimental wound with a diameter of 5 mm was formed in all rats. On days 3, and 21, five rats (n=5) in each group were randomly selected. The depicted fasting glucose data represent the mean and standard deviation. The Independent Samples T-test was used to compare the control and HFD groups on the 28th day. ANOVA was used for comparing the results among different treatment groups on day 56.

**Figure S4. Body Weight Change of of Rats in Various Treatment Groups in the Diabetic Burn Healing Model.** Groups of 20 Wistar albino rats were treated with i.p injections of RJX (1.25 mL/kg and/or 2.5 mL/kg), or vehicle (NS). Except for untreated control rats (Control), each rat was fed a high-fat diet (HFD) for 4 weeks and injected a single dose of streptozotocin (STZ, 45 mg/kg i.p.) to induce diabetes (DM). At the end of 4 weeks, an experimental burn wound with a 1 cm diameter was formed in all rats. On days 3, 7, 14, and 21, five rats in each group were randomly selected. The depicted fasting glucose data represent the mean and standard deviation. The Independent Samples T-test was used to compare the control and HFD groups on the 28th day. ANOVA was used for comparing the results among different treatment groups on day 56.

Tabular Presentation of Fasting Glucose Levels

| Groups | Time (days) | | | | *p-*value |
| --- | --- | --- | --- | --- | --- |
|  | 3 | 7 | 14 | 21 |  |
| Control | 5.71±0.61^b^ | 5.84±0.44^b^ | 5.91±0.54^c^ | 5.85±0.59^c^ | 0.949 |
| DM+NS | 25.09±1.29^a^ | 25.76±1.82^a^ | 25.00±1.94^a^ | 25.83±2.73^a^ | 0.870 |
| DM+RJX 1.25 mL/kg | 24.90±1.07^Aa^ | 24.46±1.19^ABa^ | 22.78±0.47^Bb^ | 21.90±1.20^Bb^ | <0.001 |
| DM+RJX 2.5 mL/kg | 25.19±0.82^Aa^ | 23.91±1.14^Aa^ | 21.38±0.58^Bb^ | 19.84±0.63^Cb^ | <0.001 |
| *p-*value | <0.001 | <0.001 | <0.001 | <0.001 |  |
|  | | | | | |

**Figure S5A. Blood Glucose Levels of Rats in Various Treatment Groups in the Diabetic Wound Healing Model.** Groups of 20 Wistar albino rats were treated with i.p injections of RJX (1.25 mL/kg and/or 2.5 mL/kg), or vehicle (NS). Except for untreated control rats (Control), each rat was fed a high-fat diet (HFD) for 4 weeks and injected a single dose of streptozotocin (STZ, 45 mg/kg i.p.) to induce diabetes (DM). Blood glucose levels were monitored throughout the study period. At the end of 4 weeks of HFD, an experimental wound with a diameter of 5 mm was formed in all rats. On days 3, 7, 14, and 21, five rats (n=5) in each group were randomly selected. The depicted fasting glucose data represent the mean and standard deviation. ^A,B,C^ Means in a row with different superscripts are statistically different (*p*<0.05). ^a,b,c^ Means in a column with different superscripts are statistically different (*p*<0.05). ANOVA and Tukey’s post-hoc test were used for comparing the results among different treatment groups or different times.

Tabular Presentation of Serum Insulin Levels

| Groups | Time (days) | | *p-*value* |
| --- | --- | --- | --- |
|  | 3 | 21 |  |
| Control | 17.28±2.18^a^ | 16.56±1.84^a^ | 0.584 |
| DM+NS | 7.81±0.42^b^ | 7.62±0.54^c^ | 0.597 |
| DM+RJX 1.25 ip | 7.91±0.51^b^ | 8.68±0.48^c^ | 0.040 |
| DM+RJX 2.5 ip | 8.01±0.66^b^ | 10.28±0.62^b^ | 0.001 |
| *p-*value | >0.001 | >0.001 |  |
|  | | | |

**Figure S5B. Serum Insulin Levels of Rats in Different Treatment Groups in in the Diabetic Wound Healing Model.** Groups of 20 Wistar albino rats were treated with i.p injections of RJX (1.25 mL/kg or 2.5 mL/kg) or vehicle (NS). Except for untreated control rats (Control), each rat was fed a high-fat diet (HFD) for 4 weeks and injected a single dose of streptozotocin (STZ, 45 mg/kg i.p.) to induce diabetes (DM). At the end of 4 weeks, an experimental wound with a diameter of 5 mm was formed in all rats. On days 3, and 21, five rats (n=5) in each group were randomly selected. The depicted fasting glucose data represent the mean and standard deviation. ^a,b,c^ Means in a column with different superscripts are statistically different (*p*<0.05). ANOVA and Tukey’s post-hoc test were used for comparing the results among different treatment groups. * Independent Samples T-test was used for pairwise comparing the results among different time (Day 3 vs Day 21) groups.

Tabular Presentation of Fasting Glucose Levels

| Groups | Time (days) | | | | *p-*value |
| --- | --- | --- | --- | --- | --- |
|  | 3 | 7 | 14 | 21 |  |
| Control | 5.53±0.50^b^ | 5.49±0.37^b^ | 5.64±0.50^c^ | 5.81±0.27^d^ | 0.654 |
| DM+NS | 25.97±1.32^a^ | 25.88±1.10^a^ | 26.18±0.58^a^ | 26.39±0.59^a^ | 0.843 |
| DM+RJX 1.25 mL/kg | 25.78±2.45^a^ | 25.46±1.48^a^ | 23.78±1.15^ab^ | 23.22±0.86^b^ | 0.059 |
| DM+RJX 2.5 mL/kg | 25.54±1.19^Aa^ | 24.93±1.08^ABa^ | 22.64±2.34^BCb^ | 20.74±0.65^Cc^ | <0.001 |
| *p-*value | <0.001 | <0.001 | <0.001 | <0.001 |  |

**Figure S6A. Blood Glucose Levels of Rats in Various Treatment Groups in the Diabetic Burn Healing Model.** Groups of 20 Wistar albino rats were treated with i.p injections of RJX (1.25 mL/kg and/or 2.5 mL/kg), or vehicle (NS). Except for untreated control rats (Control), each rat was fed a high-fat diet (HFD) for 4 weeks and injected a single dose of streptozotocin (STZ, 45 mg/kg i.p.) to induce diabetes (DM). At the end of 4 weeks, an experimental burn wound with a 1 cm diameter was formed in all rats. On days 3, 7, 14, and 21, five rats in each group were randomly selected. The depicted fasting glucose data represent the mean and standard deviation. ^A,B,C^ Means in a row with different superscripts are statistically different (*p*<0.05). ^a,b,c, d^ Means in a column with different superscripts are statistically different (*p*<0.05). ANOVA and Tukey’s post-hoc test were used for comparing the results among different treatment groups or different times.

Tabular Presentation of Serum Insulin Levels

| Groups | Time (days) | | *p-*value* |
| --- | --- | --- | --- |
|  | 3 | 21 |  |
| Control | 15.64±1.54^a^ | 15.44±1.86^a^ | 0.864 |
| DM+NS | 8.86±0.54^b^ | 7.46±0.41^c^ | 0.002 |
| DM+RJX 1.25 mL/kg | 8.82±0.89^b^ | 9.38±0.66^b^ | 0.289 |
| DM+RJX 2.5 mL/kg | 8.49±0.95^b^ | 10.90±0.31^b^ | <0.001 |
| *p-*value | <0.001 | <0.001 |  |

**Figure S6B. Fasting Serum Insulin Levels of Rats in Various Treatment Groups in the Diabetic Burn Healing Model.** Groups of 20 Wistar albino rats were treated with i.p injections of RJX (1.25 mL/kg and/or 2.5 mL/kg), or vehicle (NS). Except for untreated control rats (Control), each rat was fed a high-fat diet (HFD) for 4 weeks and injected a single dose of streptozotocin (STZ, 45 mg/kg i.p.) to induce diabetes (DM). At the end of 4 weeks, an experimental burn wound with a 1 cm diameter was formed in all rats. On days 3 and 21, five rats in each group were randomly selected. The depicted fasting glucose data represent the mean and standard deviation.

^a,b,c^ Means in a column with different superscripts are statistically different (*p*<0.05). ANOVA and Tukey’s post-hoc test were used for comparing the results among different treatment groups. * Independent Samples T-test was used for pairwise comparing the results among different time (Day 3 vs Day 21) groups.

**
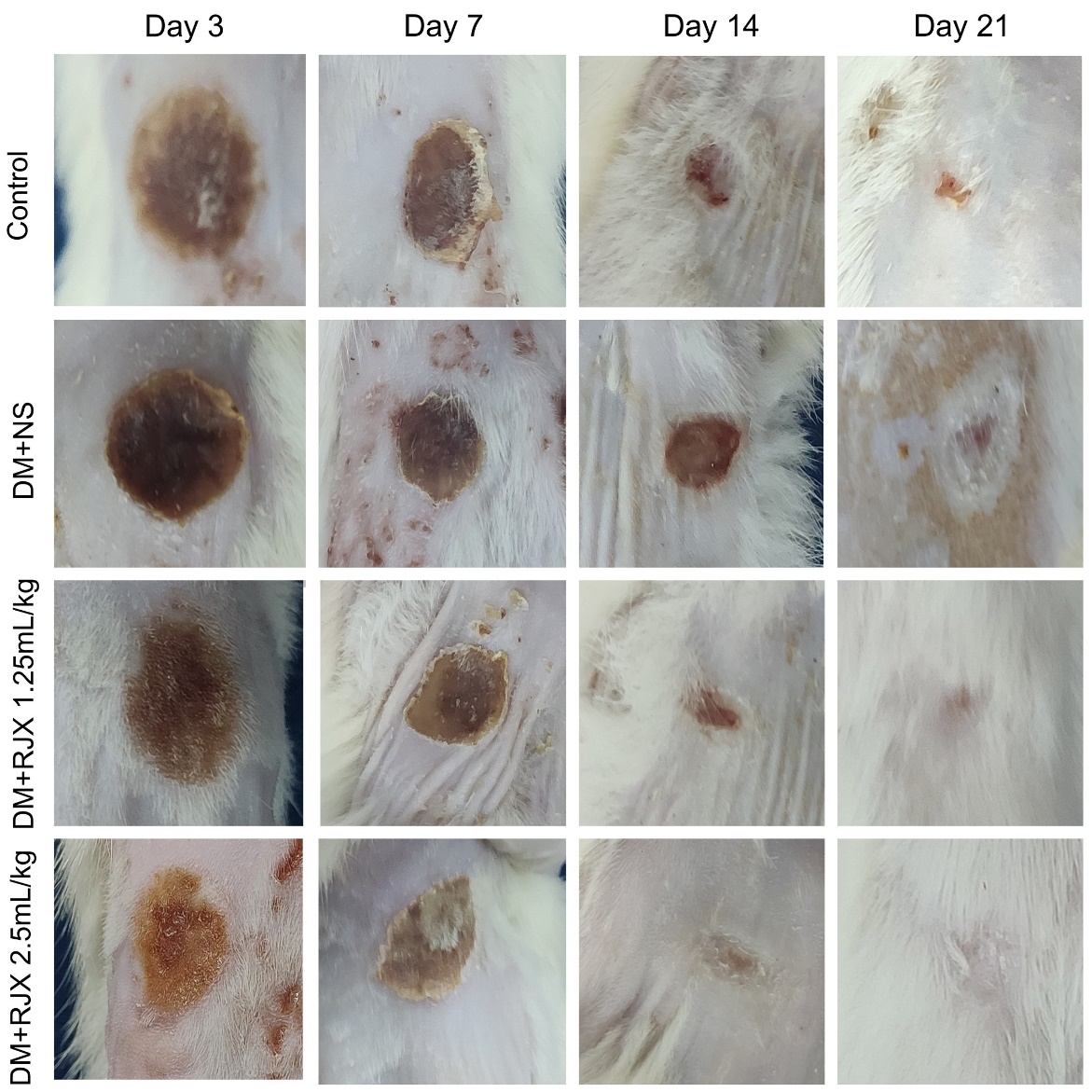
**

**Figure S7. The Effects of Rejuveinix (RJX) on Macroscopic Changes in Burn Wounds of Diabetic Rats.** Groups of 20 Wistar albino rats were treated with i.p injections of RJX (1.25 mL/kg and/or 2.5 mL/kg) or vehicle (NS). Except for untreated control rats (Control), each rat was fed a high-fat diet (HFD) for 4 weeks and injected a single dose of streptozotocin (STZ, 45 mg/kg i.p.) to induced diabetes (DM). An experimental burn wound with a 1 cm diameter was induced in all rats, as described in Materials and Methods. Wound healing was monitored macroscopically by taking photographs using a digital camera (Nikon D90, Tokyo, Japan) at multiple time points post-skin burn (days 3, 7, 14, and 21). Depicted are burn area photographs from representative rats. An accurate quantitative comparison of the wound area among different treatment groups was not feasible macroscopically due to necrotic tissue covering the burn surface of several rats at early time points and irregular scar formation at later time points.


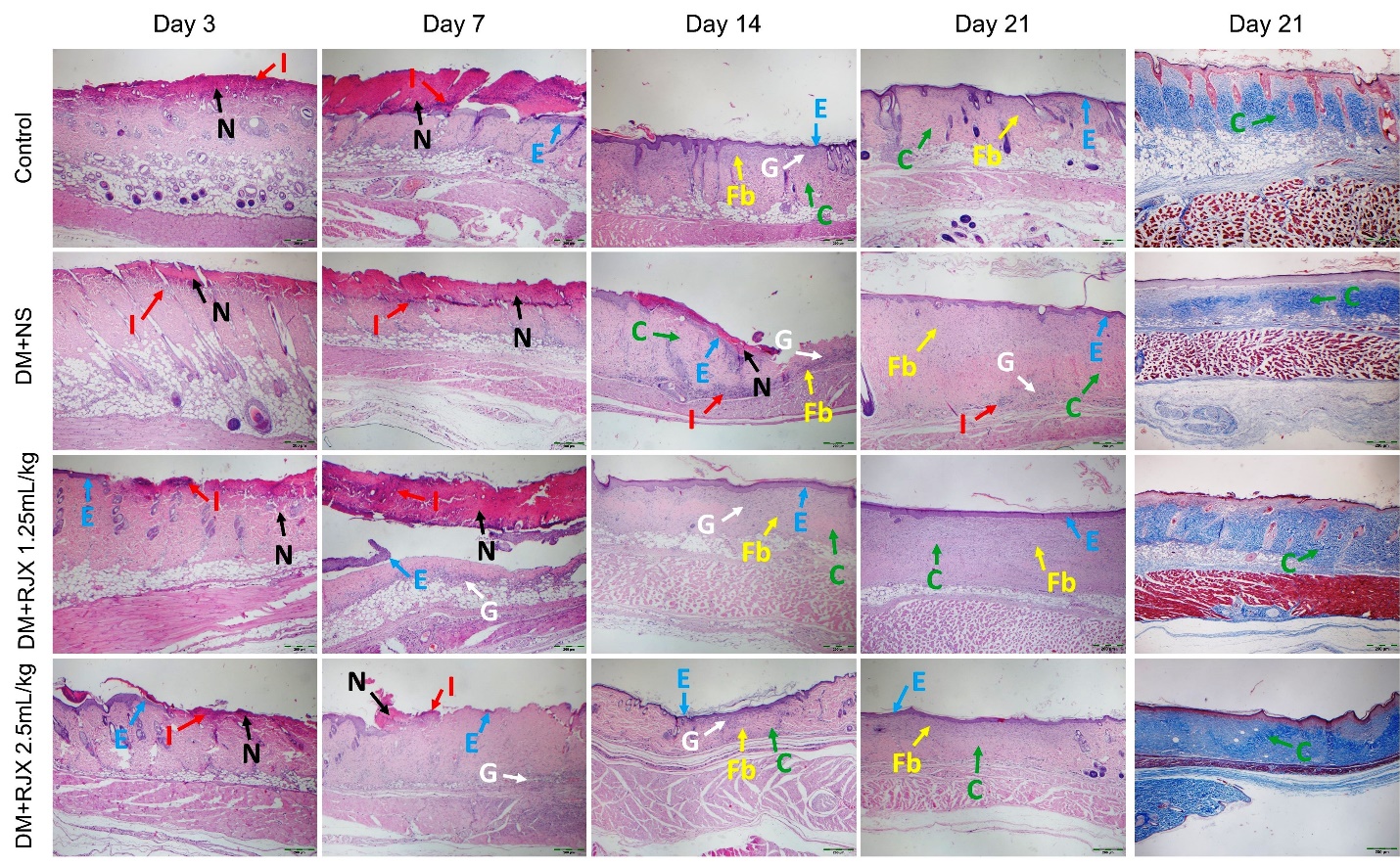


**Figure S8. The Effects of Rejuveinix (RJX) on Histopathological Changes in Burn Wounds of Diabetic Rats.** Groups of 20 Wistar albino rats were treated with i.p injections of RJX (1.25 mL/kg and/or 2.5 mL/kg) or vehicle (NS). Except for untreated control rats (Control), each rat was fed a high-fat diet (HFD) for 4 weeks and injected a single dose of streptozotocin (STZ, 45 mg/kg i.p.) to induced diabetes (DM). At the end of 4 weeks, an experimental burn wound with a 1 cm diameter was formed in all rats. On days 3, 7, 14, and 21, five rats in each group were randomly selected. Columns 1-4: Black arrow (N): Necrosis; Red arrow (I): Inflammation; Blue arrow (E): Re-epithelialization; White arrow (G): Granulation; Green arrow (C): Collagen; Yellow arrow (Fb): Fibrosis. H&E X40. Column 5: Amplified Collagen Deposition in burn wound of RJX-treated Diabetic Rats on Day 21. Green arrow (C): Collagen. Masson's trichrome staining. X40.

E. Tabular Presentation of Burn Wound Scores

| Groups | Time (days) | | | | *p-*value |
| --- | --- | --- | --- | --- | --- |
|  | 3 | 7 | 14 | 21 |  |
| Control | 0.60±0.55^B^ | 1.40±0.55^B^ | 4.40±0.55^Aab^ | 5.20±0.45^A^ | <0.001 |
| DM+NS | 0.20±0.45^D^ | 1.20±0.45^C^ | 3.40±0.55^Bb^ | 4.60±0.55^A^ | <0.001 |
| DM+RJX 1.25 mL/kg | 0.60±0.55^B^ | 1.40±0.55^B^ | 4.60±0.55^Aa^ | 5.40±0.55^A^ | <0.001 |
| DM+RJX 2.5 mL/kg | 0.60±0.55^B^ | 1.40±0.55^B^ | 4.60±0.55^Aa^ | 5.60±0.55^A^ | <0.001 |
| *p-*value | 0.516 | 0.890 | 0.033 | 0.072 |  |

**Figure S9. The Effects of Rejuveinix (RJX) on Histopathological Scores of Burn Wounds in Diabetic Rats.** Groups of 20 Wistar albino rats were treated with i.p injections of RJX (1.25 mL/kg and/or 2.5 mL/kg), or vehicle (NS). Except for untreated control rats (Control), each rat was fed a high-fat diet (HFD) for 4 weeks and injected a single dose of streptozotocin (STZ, 45 mg/kg i.p.) to induce diabetes (DM). At the end of 4 weeks, an experimental burn wound with a 1 cm diameter was formed in all rats. On days 3, 7, 14, and 21, five rats in each group were randomly selected. The depicted histopathological score data represent the mean and standard deviation. ^A,B,C^ Means in a row with different superscripts are statistically different (*p*<0.05). ^a,b,c^ Means in a column with different superscripts are statistically different (*p*<0.05). Kruskal Wallis and Mann Whitney U test were used for comparing the results among different treatment groups or different times.

**Figure S10. The Effects of Rejuveinix (RJX) on Hydroxyproline (Panel A), Total Protein (Panel B), Malondialdehyde (MDA, Panel C), and Superoxide Dismutase (SOD, Panel D) Levels in Burn Wounds of Diabetic Rats.** Groups of 20 Wistar albino rats were treated with i.p injections of RJX (1.25 mL/kg or 2.5 mL/kg) or vehicle (NS). Except for untreated control rats (Control), each rat was fed a high-fat diet (HFD) for 4 weeks and injected a single dose of streptozotocin (STZ, 45 mg/kg i.p.) to induce diabetes (DM). At the end of 4 weeks, an experimental burn wound with a 1 cm diameter was induced in all rats. On day 21, five rats in each group were sacrificed for biomarker studies. The depicted Whisker plots represent the median and min-max values. ANOVA and Tukey’s post-hoc test were used for comparing the results among different treatment groups. Statistical significance between groups is shown by * p<0.05; ** p<0.01; *** p<0.001; **** p<0.0001 as compared to control group, and ## p<0.01; ### p<0.001; #### p<0.0001 as compared to DM+NS group, and + *p*<0.05; ++ *p*<0.01; ++++ *p*<0.0001 pairwise comparisons between the groups.
